# Use of Mental Health Apps by Breast Cancer Patients and Their Caregivers in the United States: Protocol for a Pilot Pre-Post Study

**DOI:** 10.2196/11452

**Published:** 2019-01-14

**Authors:** Philip I Chow, Shayna L Showalter, Matthew S Gerber, Erin Kennedy, David R Brenin, Anneke T Schroen, David C Mohr, Emily G Lattie, Wendy F Cohn

**Affiliations:** 1 Center for Behavioral Health and Technology Department of Psychiatry and Neurobehavioral Sciences University of Virginia Charlottesville, VA United States; 2 Department of Surgery University of Virginia Charlottesville, VA United States; 3 Department of Systems Engineering University of Virginia Charlottesville, VA United States; 4 Department of Public Health Sciences University of Virginia Charlottesville, VA United States; 5 Center for Behavioral Intervention Technologies Department of Medical Social Sciences and Psychiatry and Behavioral Sciences Northwestern University Chicago, IL United States

**Keywords:** cancer, caregivers, mental health, mHealth

## Abstract

**Background:**

Over one-third of cancer patients experience clinically significant mental distress, and distress in caregivers can exceed that of the cancer patients for whom they care. There is an urgent need to identify scalable and cost-efficient ways of delivering mental health interventions to cancer patients and their loved ones.

**Objective:**

The aim of this study is to describe the protocol to pilot a mobile app–based mental health intervention in breast cancer patients and caregivers.

**Methods:**

The IntelliCare mental health apps are grounded in evidence-based research in psychology. They have not been examined in cancer populations. This pilot study will adopt a within-subject, pre-post study design to inform a potential phase III randomized controlled trial. A target sample of 50 individuals (with roughly equal numbers of patients and caregivers) at least 18 years of age and fluent in English will be recruited at a US National Cancer Institute designated clinical cancer center. Consent will be obtained in writing and a mobile phone will be provided if needed. Self-report surveys assessing mental health outcomes will be administered at a baseline session and after a 7-week intervention. Before using the apps, participants will receive a 30-min coaching call to explain their purpose and function. A 10-min coaching call 3 weeks later will check on user progress and address questions or barriers to use. Self-report and semistructured interviews with participants at the end of the study period will focus on user experience and suggestions for improving the apps and coaching in future studies.

**Results:**

This study is ongoing, and recruitment will be completed by the end of 2018.

**Conclusions:**

Results from this study will inform how scalable mobile phone-delivered programs can be used to support breast cancer patients and their loved ones.

**Trial Registration:**

ClinicalTrials.gov NCT03488745; https://clinicaltrials.gov/ct2/show/NCT03488745

**International Registered Report Identifier (IRRID):**

DERR1-10.2196/11452

## Introduction

### Background

In the United States, an estimated 266,120 new cases of invasive breast cancer are expected to be diagnosed in 2018 [1]. Breast cancer is the most common form of cancer in women and the second leading cause of cancer-related deaths in women. In the United States, over 40% of newly diagnosed breast cancer patients report clinically significant distress [2]. Cancer affects not only patients but also their caregivers, which can include a partner, relative, or friend. Responsibilities directly (eg, coordinating care) and indirectly (eg, providing emotional support) linked to their loved one’s care leave caregivers at high risk for burnout [3]. However, despite levels of psychological distress that can exceed those of the cancer patients for whom they care [4], caregivers of cancer patients remain an underserved, yet vulnerable, population. Thus, there is an urgent need to identify ways to provide supportive care to both cancer patients and their loved ones.

### Mobile Interventions for Cancer Populations

Although distress screening has become a standard practice for many cancer programs [5], distress intervention through mobile technology remains an important need in the cancer community [6]. Community and health care organizations are important providers of support services for both cancer patients and their caregivers. Many of them are beginning to provide services through virtual means such as the phone and internet. However, existing models of psychosocial intervention that are heavily reliant on human support are costly and not readily scalable to large populations. For example, on-demand phone helplines need to be constantly staffed by nurses or mental health professionals and are limited in their ability to address the needs of a large and growing cancer population in the United States. Given that over 77% of American adults own a mobile phone [7], it is an ideal platform from which to deliver brief, empirically supported interventions to anyone that needs them. Mental health apps are easily scalable and can provide tailored interventions when and where they are most needed.

### Limitations of Prior Work

Despite the promise of mobile phone mental health apps, significant issues need to be addressed before making them widely available to cancer populations. Although researchers are increasingly examining the efficacy and effectiveness of mental health apps, reviews have found that few publicly available apps have any empirical evidence supporting them [8,9]. These reviews generally paint a bleak outlook for the presence of empirically supported mental health apps, which is negatively impacted by the proliferation of mental health apps in recent years. Although more than 10,000 mental health–related apps are available for download, the lack of thorough investigation of these apps by clinical scientists, combined with a lack of government regulatory oversight, makes many of these apps unhelpful at best and dangerous at worst [10-13]. It is, therefore, imperative to empirically validate mental health apps for the population they are intended for, such as mental health apps for cancer populations. Research has found that the majority of apps for cancer either focus on cancer-symptom monitoring or are intended to raise awareness of cancer through fundraising or promoting a charitable organization [6]. A conclusion to be made from these papers is that a dearth of mental health apps have been tested and designed for cancer populations, which served as the impetus for undertaking this study.

Most health-related apps suffer from poor usability for a variety of reasons such as requiring lengthy engagement times that do not match user preferences [14]. In reality, people use apps in short, frequent time bursts and prefer apps that support a single or limited set of tasks [15]. Providing brief and targeted interventions is particularly important for cancer patients and caregivers as the demands of cancer treatment often leave them with small pockets of time throughout the day. Importantly, pairing an app with light coaching can further increase motivation and adherence [16,17]. In contrast to the majority of support apps that do not provide human assistance, light phone coaching can increase adherence by focusing on how apps can address people’s needs and by identifying obstacles to their effective use [16,17].

### Objective of This Study

**Figure 1:**
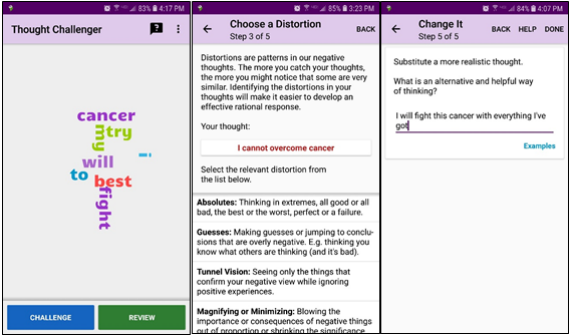
Screenshots of the Thought Challenger app as seen on a mobile phone screen.

The purpose of this study is to conduct a pilot study that uses a set of brief, targeted app-delivered interventions that promote mental health. IntelliCare is a collection of apps that use an elemental, skill-based approach to improving mental health [18]. App content is based on evidence-based approaches in cognitive behavioral therapy (CBT) as well as concepts from mindfulness and positive psychology. For example, one of the apps, Thought Challenger (see Figure 1), guides individuals through an exercise to identify and challenge negative thinking styles, a common CBT approach for anxiety and depression. Another app, Purple Chill, provides users with mindfulness audios that can be accessed at any time. Users can download up to 12 publicly available intervention apps, each of which targets a specific aspect of mental health and well-being (eg, identifying maladaptive thoughts, promoting sleep, and increasing relaxation skills). The apps are designed to be interactive and intuitive. Users can complete many exercises (eg, identifying and challenging an unhelpful thought) in less than a minute. Exercises require few instructions to complete and are usually found on the first screen that is presented. Each IntelliCare app has a *help* feature that contains educational and technical content regarding the specific app in question. See Table 1 for a description of IntelliCare apps and their objectives.

IntelliCare apps are available on both Android and iPhone stores. Those from the general public who download the apps are free to use the apps as desired [19]. Similar to prior IntelliCare work [18], participants in this study are instructed to systematically try 1 to 2 apps per week and retain the ones that are most helpful to them. The purpose of this strategy is to gradually expose participants to all of the apps in a systematic manner, until all available apps have been tried. This mirrors face-to-face CBT in which clients are encouraged to acquire various skills through practice. Users will determine which apps to use (see Methods section). In an 8-week study with light phone coaching, over 90% of users with elevated depression and anxiety symptoms used the apps an average of 195 times, for an average length of 1 min [18]. In this initial study, IntelliCare usage was notably higher than what has been observed in other electronic health and mobile health (mHealth) intervention programs. It was also found that using the apps led to large and significant decreases in depression (Cohen *d*=1.4) and anxiety symptoms (Cohen *d*=1.2), as measured by the Patient Health Questionnaire (9-item version [20]) and the Generalized Anxiety Disorder questionnaire (7-item version [21]), respectively [18]. However, the acceptability, usability, and potential impact of IntelliCare apps in *cancer* patients and caregivers are unknown. Cancer patients and caregivers are faced with the multiple physical and emotional sequalae of cancer treatment, making them potentially unique from other populations. To design mental health apps that can benefit cancer populations, it is important to understand their preferences for using them.

The purpose of this trial was to conduct a pilot study to inform a potential phase III randomized controlled trial (RCT). Consistent with prior definitions and reasons for conducting a pilot study [22,23], the goals of this study are to (1) assess the feasibility of various components (eg, recruitment rates, retention rates, and refusal rates) that need to take place in a larger study; (2) understand and identify potential human and data optimization issues (eg, issues of managing the study in a busy clinic and identifying challenges to recruitment from doctors and nurses, whether data show too much or too little variability); and (3) examine whether participants respond to the intervention. Importantly, a pilot study is not only concerned with whether something can be done and how to proceed but includes implementing something in a way intended in part of a future study [22].

**Table 1:** Description of IntelliCare apps and their objectives.

App name	Objective
Aspire	Promote awareness of and striving toward personal goals and values. Helps users identify their values and keep track of their progress.
Day to Day	Promote knowledge about ways to bolster mood. Users receive a daily stream of knowledge tidbits and are prompted to build on a theme every day (eg, cultivate gratitude and problem solve).
Daily Feats	Promotes goal setting and attainment. An in-app built calendar allows users to track their successes and identify new tasks to complete.
Worry Knot	Promotes knowledge about worry and provides an interactive exercise to decrease worry. The app also tracks the user’s progress and provides tailored feedback on ways to distract oneself from worrying thoughts.
Social Force	Encourages users to identify supportive individuals in their life. The app prompts users to reach out to these people for encouragement.
My Mantra	Increases self-efficacy and a positive perspective of oneself. The app prompts users to come up with personal mantras and to construct personalized photo albums that serve as reminders of these mantras.
Thought Challenger	Increases the ability to identify and challenge negative thinking patterns. Guides users through a cognitive restructuring exercise and tracks the output of past exercises.
iCope	Promotes coping and positive reinforcement by having users write and send themselves encouraging messages when they are most needed.
Purple Chill	Increases relaxation skills by providing a library of mindfulness and guided meditation audio files.
MoveMe	Promotes mood through physical activity. The app prompts users to schedule exercises throughout the day or week and provides instructional videos and lessons to increase motivation to exercise.
Slumber Time	Promotes healthy sleeping by prompting users to keep an active sleep diary. The app also provides a checklist of things to do before bedtime to promote healthy sleep habits.
Boost Me	Promotes positive mood by having users schedule positive activities throughout the day. A mood tracker allows users to see their progress and the impact of different activities on their mood.

## Methods

### Study Design

This single-group, 7-week, pre- and posttest pilot study will provide IntelliCare apps to a sample of breast cancer patients and their caregivers in the United States. A mixed-methods approach using self-report measures and qualitative interviews will be used to evaluate user satisfaction and potential for adoption in a larger and more diverse cancer population. In addition, because the apps used in this study are not tailored for breast cancer populations, qualitative interviews will yield crucial feedback to determine what changes to the IntelliCare apps can be made in a larger trial. Passively collected app usage data (ie, app launches and app session duration) will inform our understanding of engagement with the apps among cancer patients and caregivers. The decision to use a 7-week duration was based on the duration of brief face-to-face psychotherapy (typically 6-8 weeks) as well as prior reviews of mHealth studies, finding that the duration of app interventions range between 6 days and 8 weeks [8].

### Participants

To limit barriers to entry, inclusion criteria are limited to the following: (1) breast cancer patient or informal caregiver (ie, not receiving compensation for providing care); (2) at least 18 years of age; (3) proficient in English at a sixth grade level; and (4) has a mobile phone or is willing to carry one around if provided. Participants are not required to have a minimum level of familiarity with mobile devices or technology. Participants will be eligible to receive a US $50 gift card for providing user feedback at the end of the study. Study procedures, including the coaching protocol and access to IntelliCare apps, will be identical for both breast cancer patients and caregivers.

A target sample size of 50 (25 patients and 25 caregivers) was chosen, given the exploratory nature of this study. Recruitment of participants will occur in a small breast surgical oncology clinic. Clinics are held on 4 out of 5 weekdays, and the 3 surgical oncologists do not have overlapping clinic times. The recruitment goals were influenced by several factors. First, the patients seen in this clinic are generally early in the breast cancer diagnostic pathway and although it is a time of need for the type of mental health support these apps can provide, it is also a time when it may not be appropriate to recruit them to a study that requires an immediate face-to-face consent and app download process. Second, the logistics of clinic flow, space, and time constraints also influence the pace of recruitment. Overall, 1 to 2 participants per week became the targeted number, which over the course of 29 weeks of active recruitment would yield a sample size between 29 and 58 consented participants.

The primary objective of this study is to inform the feasibility of a larger randomized trial in a clinical setting [22]. Data on usability and user experience from this sample will enable researchers to make iterative changes for future studies. A secondary objective of this study is to provide effect size estimates for a future trial and to pilot the data analyses. This will allow us to explore potential data analytic issues that might arise in a large trial such as missingness and skewness. Within-group analyses will be performed for both breast cancer patients and caregivers (see Data Analysis section). For reference, an effect size of *d=* 1.4 for change in depression and anxiety symptoms was found in a prior noncancer sample [18] at 80% power. However, it may not be appropriate to generalize an effect size from a symptomatic depressed sample to an unselected cancer population. For general reference, the smallest effect size that can be detected with a sample size of 25, using paired *t* tests with 80% power and alpha of .05, is *d*=0.58, whereas the smallest effect sizes that can be detected with a sample size of 35, using paired *t* tests with 80% power and alpha of .05, is *d*=0.49 [24].

### Materials

Participants will use their own personal mobile phone (Android or iPhone). Some individuals may not own a mobile phone or have an appropriate mobile phone plan that enables downloading and using a native mobile phone app. To address these issues, those who do not own a mobile phone or have an incompatible device will be provided with a Samsung S7 Android phone with an unlimited data plan. Those who are provided a phone will be able to use it for nonstudy purposes. A concerted effort was made to include both Android and iPhone users in the study, given the differences between users of these platforms in some prior work [25]. All IntelliCare apps are currently available for Android users, and a subset of iPhone apps are available (as of March 2018), although more are planned for release.

### Recruitment Procedure

Breast cancer patients and their caregivers will be recruited from a breast care clinic. Surgical oncologists will help to identify potential participants, who will be introduced to the study during a normal scheduled visit. Surgical oncologists will offer a study flyer to breast cancer patients and their caregivers during a normal scheduled visit. Patients and caregivers who express an interest in learning about the study will then speak to a research staff member, who will provide more details about the study and answer any questions. If an eligible patient or caregiver expresses interest in participating in the study, he or she will be led through the consenting process by a research staff member. Research staff will describe the aims of the study, introduce the IntelliCare apps, and review the study timeline. Infographics will serve as visual aids to improve understanding of the study components and timeline. Individuals who provide written consent will schedule a 30-min coaching call to take place sometime within the next 10 days. They will also be guided to download the apps but will be told not to open them until the coaching call. Downloading the apps at the end of the consenting process will allow coaching calls to be kept to within the allotted 30-min time frame. Participants will also have the option to download a separate app (ie, not part of the IntelliCare app suite) that passively collects location and movement data from their phone’s sensors. This app, Sensus, was developed by University of Virginia researchers [26] and has been used to collect location and movement data in college student samples [27]. Data collected from this app will be used in exploratory analyses to determine whether it is possible to identify behavioral markers of mood and well-being. For example, in a prior study of college students, it was found that the amount of time spent at home was associated with a higher level of anxiety and that more negative affect was linked to a longer homestay duration [27]. Participants will be asked to spend 10 to 15 min to complete measures that assess depression and anxiety symptoms, physical and social functioning, and subjective well-being. Measures will be completed via the Web using Qualtrics Survey Platform through a desktop or laptop computer. Recruitment will cease if the target enrollment is met or funding expires at the end of 2018. The same research staff members are each responsible for consenting participants, conducting coaching calls, and collecting feedback from participants.

### Phone Coaching

A coaching protocol was developed based on the Efficiency Model of behavioral intervention technology support [16] and supportive accountability [17]. A similar coaching protocol has been implemented in a prior study of the IntelliCare apps [18]. The primary aims of coaching are to address usability issues, increase engagement with the apps, promote fit by assessing participants’ needs, promote knowledge acquisition of the skills found in the apps, and encourage implementation of the skills in participants’ lives. In keeping with the prior study of IntelliCare [18], coaches are instructed to focus on app-related issues and to refrain from doing more traditional counseling with participants. An initial 30-min coaching call will focus on orienting participants to downloading and using the apps, setting expectations of the coach’s role, assessing how the apps may meet participants’ needs, and building rapport. Participants will also be told that they can contact coaches at any time with any app-related questions. Participants who contact coaches for crisis management will be connected with a nearby mental health service provider. Any participant inquiries will receive a response within 1 working day. Following the initial coaching call, participants will receive a short message service (SMS) text message (via Qualtrics Survey Platform SMS tool) every week to remind them to download and try 1 to 2 new IntelliCare apps. Coaches have a bachelor’s degree (not in counseling) and are trained and monitored by the lead author (PC), who has a PhD in clinical and community psychology and over 8 years of experience in conducting psychological assessments and psychotherapy. Coaches received a detailed coaching manual and will attend weekly supervision meetings throughout the duration of the study.

To encourage engagement with the apps, coaches will refrain from making explicit recommendations regarding which apps to use and how often to use them. Instead, following prior work [18], coaches will instruct participants to review the apps, remind them of their needs and goals, and ultimately allow participants to make their own decisions regarding which apps to use. If participants are resistant to making their own decision regarding which apps to use, coaches will be permitted to give recommendations. Precautions were made to help ensure that coaching calls focus on the IntelliCare apps. Specifically, coaches are provided a detailed and scripted coaching manual that they are told to follow closely. Coaches received weekly training and engaged in role playing exercises with the lead author (PC) on how to conduct coaching calls. Finally, a 10-min phone call 3 weeks after the initial coaching call will serve as a check-in to make sure that participants have a clear understanding of the app program and to answer any lingering questions. All described components of this study have been approved by the University of Virginia institutional review board for Health Sciences Research.

### Measures and Outcomes

#### Primary Objective and Measures

Because a significant barrier to conducting this study is the enrollment of participants in a busy clinical setting, we will consider the study feasible if (1) we are able to recruit 1 to 2 participants per week from a single clinic over 29 weeks; (2) complete follow-up in at least 50% of all recruited subjects; and (3) observe a median app launch rate of around 3.0, as found in prior work [18]. Participants will be asked to provide feedback on the apps and coaching at the end of the study period. The Usability, Satisfaction, and Ease of use (USE [28]) short form scale will be used to examine usability of the IntelliCare apps. It is composed of items that assess user experience (eg, “I would recommend it to a friend,” “It is easy to learn to use it,” and “It is simply to use”). Items are scored on a 7-point Likert scale (1=strong disagree and 7=strongly agree). The USE measure is a validated scale that is commonly used to evaluate user experience of digital interventions. Participants will be asked to describe the most positive and negative aspects of the apps and which apps were most and least helpful and why. If participants stopped using the apps, they will be asked to comment on why and barriers to using the apps.

Participants will also be asked to provide open-ended feedback. Research staff will conduct telephone interviews with participants, which will cover the following topics related to using the apps: general impressions, design quality, technical needs, and design suggestions to promote app implementation and usage. In addition, participants will be asked to provide feedback on the following aspects of phone coaching: general experience with coaches, usefulness of coaching, additional or unmet coaching needs, and suggestions to improve the coaching experience. Thematic analysis will be used to analyze qualitative data gathered from interviews. Data yielded from this mixed-methods approach will be used to make improvements to the apps and phone coaching in future work.

#### Secondary Objectives and Measures

Several measures will be administered at baseline and after 7 weeks. Demographic and background variables (eg, age, gender, and race or ethnicity) will be collected at the baseline session. Disease variables (eg, cancer site, stage, and treatments) will also be collected from the patient’s electronic medical records. Measures will be administered and completed via a secure data collection website (Qualtrics Survey Platform). Data are stored in a secure database that is only accessible to study personnel to ensure confidentiality.

Depression symptoms will be assessed with the 4-item scale from the Patient-Report Outcomes Measurement Information System [29] 29-item profile version 2.0 (PROMIS-29 Profile v2.0). PROMIS, a US National Institutes of Health Roadmap program, provides sensitive and reliable measures of patient-reported outcomes. A goal of PROMIS is to allow organized and effective assessment of patient-reported outcomes across a range of chronic diseases. Participants are asked to report (1=never and 5=always) the degree to which they experienced various depressed states (eg, “I felt worthless” and “I felt hopeless”) over the past 7 days. Continuous anxiety symptoms will be assessed with the 4-item scale from the PROMIS-29 Profile v2.0. Participants are asked to report (1=never and 5=always) how much they have experienced different anxious states (eg, “My worries overwhelmed me” and “I felt fearful”) over the past 7 days.

The Patient Health Questionnaire-4 (PHQ-4; [30]) is widely used in cancer settings as a brief screener of general distress and symptom burden [30] and is well validated in both general and clinical samples [30,31]. The PHQ-4 will be administered to examine whether using mental health apps leads to a clinically significant decrease in general distress and overall symptom burden and will be used to classify individuals based on the severity of their mood symptoms at baseline and postassessment. Individuals are asked to rate (0=not at all and 3=nearly every day) the degree to which they have experienced different states (eg, “Little interest or please in doing things”) over the past 2 weeks. Scores range from 0 to 12. A score of 6 to 8 indicates moderate mood symptoms, whereas a score of 9 and higher indicates severe mood symptoms. Symptoms assessed by the PHQ-4 and PROMIS anxiety and depression subscales (of PROMIS-29) will be used to obtain estimates of the treatment effects and the variances of treatment effects for a larger trial.

Several measures will be administered to both patients and caregivers. Life meaning will be assessed with the 4-item PROMIS [29] Life Meaning/Purpose scale. Participants are asked to report (1=not at all and 5=very much) the degree to which they agree with 4 statements (eg, “My life has meaning” and “I have a clear sense of direction in life”). Sleep quality will be assessed with the 4-item PROMIS [29] Sleep Disturbance scale from the PROMIS-29 Profile v2.0. Participants are asked to report (1=very poor and 5=very good) on their sleep quality over the last 7 days. They are also asked to report (1=not at all and 5=very much) the degree to which they experienced sleep difficulties (eg, “I had difficulty falling asleep”) over the last 7 days. Patients and caregivers will also complete a measure of health care utilization. Patients will be asked whether they visited the emergency department over the past 2 months, whether any of these visits were related to side effects from cancer treatment, whether they missed a scheduled appointment for cancer treatment, and whether they have used cancer support services in the past 2 months. Caregivers will be asked whether they visited the emergency department over the past 2 months, how many times they visited a primary care doctor for anything other than routine care, and whether they have used cancer support services in the past 2 months.

Several additional scales will be administered to patients. Physical functioning will be assessed with the 4-item PROMIS [29] Physical Health scale from the PROMIS-29 Profile v2.0. Participants are asked to report (1=unable to do and 5=without any difficulty) the degree to which they are able to perform 4 activities (eg, “Are you able to go for a walk of at least 15 minutes?” and “Are you able to run errands and shop?”). Engagement in social activities will be assessed with the 4-item PROMIS [29] Ability to Participate in Social Roles and Activities scale from the PROMIS-29 Profile v2.0. Participants are asked to report (1=never and 5=always) the degree to which they agree with 4 statements (eg, “I have trouble doing all of my regular leisure activities with others” and “I have trouble doing all of the family activities that I want to”). Fatigue will be assessed with the 4-item PROMIS [29] Fatigue scale from the PROMIS-29 Profile v2.0. Participants are asked to report (1=not at all and 5=very much) the degree to which they agree with 4 statements and questions (eg, “I feel fatigued” and “How run-down did you feel on average?”) as it pertains to the prior 7 days. Finally, pain interference will be assessed with the 4-item PROMIS [29] Pain Interference scale from the PROMIS-29 Profile v2.0. Participants are asked to respond (1=not at all and 5=very much) to questions assessing pain interference in daily life (eg, “How much did pain interfere with your day to day activities?” and “How much did pain interfere with your household chores?”) as it pertains to the prior 7 days. Finally, participants will respond to a single item assessing pain level over the past 7 days, on a 0 (no pain) to 10 (worst pain imaginable) scale.

Caregivers will be administered the 21-item Caregiver Self-Efficacy scale (CaSES [32]), which was developed to measure self-efficacy in informal cancer caregivers. The CaSES was found to have good validity and reliability in a large sample of caregivers [32]. Items assess caregivers’ perceptions of their duties and capabilities (eg, “I can be positive when I need to be,” “I can continue to provide care when I feel scared,” and “I have the ability to talk openly with the person I care for”) and are scored on a 4-point scale (0=not at all confident and 4=very confident).

Finally, IntelliCare app use data will be collected passively. Specifically, engagement will be ascertained from the number of app launches, defined as a user-initiated event after at least 5 min of no activity [18]. The duration of individual app use sessions will also be used to reflect engagement and is defined as the length between an app launch at the last event in that session.

To understand the preliminary impact of IntelliCare on daily mood, social functioning, and health behavior during the study, patients and caregivers will respond to a short survey every week throughout the study period via the Web. Surveys will be delivered using the Qualtrics Survey Platform SMS tool. Participants will receive an SMS text message on their phone at 8 pm. An embedded link within the SMS text message will automatically connect participants to a secure Qualtrics Survey Platform Web page containing survey items. Weekly surveys are each expected to take 1 to 2 min to complete.

All participants will be asked to report (1=very negative and 5=very positive) how they have felt over the past week and how they expect to feel the following week. They will also be asked about the following behaviors or activities over the past week: how well they have managed negative feelings, how much they have used alcohol or tobacco to cope with negative feelings, amount of physical pain experienced, how connected they felt to family and friends, how much support they received from loved ones, how much support they were able to provide to loved ones, how much anxiety they experienced, how much interest or pleasure they had in doing things, and amount of physical activity. At the end of the survey, participants are reminded to focus on trying out new IntelliCare apps for the upcoming week. They will be asked to note which specific apps they intend to use during the upcoming week.

### Data Analysis

All data will be stored in a secured server for highly sensitive data. Data will be cleaned and analyzed in statistical software packages (ie, SPSS, SAS, and R). Protocol nonadherence will be defined as individuals who fail to complete the baseline and postintervention surveys. Because this pilot study is only being conducted at a single site, a data monitoring committee was not utilized.

Quantitative data on user experience will be analyzed descriptively, to be compared with user data in existing literature. Qualitative user experience data will be reviewed for content and emerging themes through content analysis. Participants’ interview responses will be recorded by research staff. Qualitative responses will be coded and evaluated according to the general domains of (1) ways to improve the design and user interface of the apps; (2) the specific apps that were most helpful (and why); (3) the specific apps that were least helpful (and why); (4) obstacles and barriers to using the apps; and (5) ways to improve the usefulness of coaching calls. Initial coding of the data will be based on a priori domains and will be refined during the analysis process conducted by investigators. Additional themes that are identified will be defined and coded.

Due to the within-subject pre-post design, changes in outcome measures in both cancer patients and caregivers will primarily be analyzed using paired *t* tests. Descriptive statistics will primarily be used to examine whether IntelliCare app use is associated with changes in process variables (ie, mood, social functioning, and health behavior) during the study period. These analyses will be performed separately for patients and caregivers. Because the IntelliCare apps are intended as an intervention package, we will be evaluating the app suite as a whole in breast cancer patients and caregivers rather than selecting specific apps for patients and caregivers. A separate set of analyses will examine associations between changes in cancer patient and caregiver outcomes. Specifically, zero-order correlations will be computed to examine whether improvement in caregiver depression or anxiety symptoms is positively associated with improvement in patient depression or anxiety symptoms. Correlations will also be computed to examine whether improvement in caregiver self-efficacy is positively associated with improvement in patient mood symptoms. The purpose of these analyses is to obtain estimates of effect size that can be used to inform future trials. Additional discussions with cancer clinicians will be used to obtain additional information on possibility effect size and variance estimates [33]. We do not intend to perform any between-group contrast analyses (ie, between patient and caregiver or between different IntelliCare apps).

## Results

This study will run for 8 months, and recruitment will be completed by the end of 2018. The study was approved by the local university’s institutional review board. Research staff has been hired and trained, and set up has been completed to store all data on secure university servers. Recruitment commenced in March 2018. As of the end of June 2018, 17 breast cancer patients and 7 cancer caregivers have been consented. We will monitor participants’ progress and continue to recruit participants over the next 4 months or until we successfully hit our target enrollment.

## Discussion

### Principal Findings

A cancer diagnosis impacts both patients and their loved ones. Over a third of US cancer patients experience clinically significant mental distress [34]. Studies also show a high level of distress in cancer caregivers in the United States, with over 25% screening positive for depression and 35% screening positive for anxiety [35]. Unfortunately, face-to-face models of mental health care are not sufficient to meet the growing demand for mental health resources in cancer populations. Mental health and support apps may, therefore, address a critical health care gap, although few studies have evaluated the impact of mental health apps in cancer populations. To understand whether existing mental health apps can benefit cancer populations, it is important to understand their preferences for using them as well as gather information about how these apps can be tailored for cancer populations. Findings from this study will help to address this weakness in clinical care, by providing preliminary data to estimate the effect of a suite of mobile phone apps on mental health outcomes in breast cancer patients and caregivers, as well as tailor an existing intervention to better suit the needs of cancer patients and caregivers. In addition, although some studies have found unique benefits of interventions target patient-caregiver dyads [36], this study will be among the first to examine the preliminary effects of providing mental health apps to patient-caregiver dyads.

Although many apps are available on app stores, digital health technologies (which include apps, wearable sensors, and internet-delivered interventions) that are connected to a care manager in a health care setting are increasingly being used as a method of providing technology-enabled services [37,38]. Such systems can be managed by care managers, physician assistants, nurses, or other trained individuals. Although pairing an app with light coaching is a potential limitation to scalability and real-world implementation in some settings, studies such as these are critical to continue to explore the practicality and feasibility of these blended interventions (ie, interventions that combine an automated intervention with some provision of human support) as well as identify which users may or may not need human coaching or additional human support in larger trials.

Findings from systematic reviews indicate that despite a plethora of mental health apps available in app stores, only a few are empirically supported [8,9,39] and the few that target cancer populations have little to no empirical support [6]. IntelliCare apps are publicly available through both the Google Play store (for Android phones) and the App Store (for iPhones) and have been tested in individuals with clinical mood symptoms [18]. These factors create an ideal situation to conduct a pilot study in cancer patients and caregivers. Findings from this study will extend existing work on how mobile technology can be used to address mental health needs in cancer populations.

### Limitations

This study should be interpreted in light of several limitations. Because the focus of this study is to conduct a pilot study of a potential phase III trial, the sample size will be relatively small and there will be no comparison condition. Thus, it is impossible to rule out the possibility that improvement in psychosocial outcomes is because of IntelliCare apps or outside factors. We also recognize that effect sizes obtained from relatively small sample sizes can be somewhat unreliable and, thus, will be interpreted with caution [40,41]. Recruiting a larger sample size, combined with an RCT design, is an appropriate next step to understand whether using the IntelliCare apps leads to improvement compared with standard treatment. Furthermore, because this study will be recruiting individuals directly from the clinic to achieve the targeted sample size, there are few exclusion criteria that may lead to potential confounders (eg, psychiatric diagnosis). In addition, this study is conducted in a US National Cancer Institute designated clinical cancer center, and therefore, findings may have limited generalizability to settings that do not possess as many resources. Thus, we hope that data from this pilot study will inform future work that attempts to administer IntelliCare apps from those recruited from a range of clinical settings.

It should be noted that some individuals may not possess a personal mobile phone or have a data plan that allows them to participate in this study. Our strategy to help mitigate this problem will be to provide interested participants who do not have a mobile phone with an Android device and an unlimited data plan to ensure equal access to the mHealth intervention. Given that mobile phone ownership, comfort using phones, and the role of mobile phones in daily life are all interconnected, it is important to acknowledge that problems with generalizability will emerge and grow as time elapses from the end of the trial. Providing people with a mobile phone, therefore, only addresses part of this complex problem that impacts the broader field of mHealth. Similarly, future research may wish to examine barriers to scalability of mHealth interventions for cancer populations. For example, to supplement individuals with limited or prepaid phone plans, researchers may want to examine the feasibility of a partial payment plan that would enable users to upgrade their plans. What this pilot study will also provide is an estimate of the approximate percentage of individuals who require a mobile phone in future trials. Finally, to address low literacy of using technology and mobile devices, phone coaching will provide participants with any needed instructions on how to download, use, and manage the IntelliCare apps. Coaches will also be available to provide technical support as needed.

### Conclusions

Very little work has examined the potential effectiveness of mental health apps in cancer patients and their informal caregivers. This pilot study will provide preliminary data regarding the usability and acceptability of a suite of mental health apps in a sample of cancer patients and caregivers in the United States. The mixed-methods approach to gathering user feedback will provide a rich dataset that will guide improvements to the apps and coaching procedure in future studies.

## Abbreviations

CaSES: Caregiver Self-Efficacy Scale
CBT: cognitive behavioral therapy
mHealth: mobile health
PHQ-4: Patient Health Questionnaire-4
PROMIS: Patient-Report Outcomes Measurement Information System
RCT: randomized controlled trial
SMS: short message service
